# Complete genome sequence of *Streptococcus agalactiae* strain GBS85147 serotype of type Ia isolated from human oropharynx

**DOI:** 10.1186/s40793-016-0158-6

**Published:** 2016-06-03

**Authors:** Edgar Lacerda de Aguiar, Diego César Batista Mariano, Marcus Vinícius Canário Viana, Leandro de Jesus Benevides, Flávia de Souza Rocha, Letícia de Castro Oliveira, Felipe Luiz Pereira, Fernanda Alves Dorella, Carlos Augusto Gomes Leal, Alex Fiorini de Carvalho, Gabriela Silva Santos, Ana Luiza Mattos-Guaraldi, Prescilla Emy Nagao, Siomar de Castro Soares, Syed Shah Hassan, Anne Cybele Pinto, Henrique César Pereira Figueiredo, Vasco Azevedo

**Affiliations:** Laboratory of Cellular and Molecular Genetics (LGCM), Federal University of Minas Gerais, Belo Horizonte, Brazil; National Reference Laboratory for Aquatic Animal Diseases (AQUACEN), Ministry of Fisheries and Aquaculture, Federal University of Minas Gerais, Belo Horizonte, Brazil; Laboratory of Molecular Biology and Physiology of Streptococci, State University of Rio de Janeiro, Rio de Janeiro, Brazil; Faculty of Medical Sciences - State University of Rio de Janeiro, Rio de Janeiro, Brazil; Institute of Biological and Natural Sciences (ICBN)- Federal University of Triangulo Mineiro, Uberaba, Minas Gerais Brazil

**Keywords:** *Streptococcus agalactiae*, Human pathogenic bacteria, Oropharynx, Complete genome sequence, Ion torrent

## Abstract

*Streptococcus agalactiae*, also referred to as Group B *Streptococcus*, is a frequent resident of the rectovaginal tract in humans, and a major cause of neonatal infection. The pathogen can also infect adults with underlying disease, particularly the elderly and immunocompromised ones. In addition, *S. agalactiae* is a known fish pathogen, which compromises food safety and represents a zoonotic hazard. This study provides valuable structural, functional and evolutionary genomic information of a human *S. agalactiae* serotype Ia (ST-103) GBS85147 strain isolated from the oropharynx of an adult patient from Rio de Janeiro, thereby representing the first human isolate in Brazil. We used the Ion Torrent PGM platform with the 200 bp fragment library sequencing kit. The sequencing generated 578,082,183 bp, distributed among 2,973,022 reads, resulting in an approximately 246-fold mean coverage depth and was assembled using the Mira Assembler v3.9.18. The *S. agalactiae* strain GBS85147 comprises of a circular chromosome with a final genome length of 1,996,151 bp containing 1,915 protein-coding genes, 18 rRNA, 63 tRNA, 2 pseudogenes and a G + C content of 35.48 %.

**Electronic supplementary material:**

The online version of this article (doi:10.1186/s40793-016-0158-6) contains supplementary material, which is available to authorized users.

## Introduction

*Streptococcus agalactiae* is a bacterial pathogen, distributed worldwide, that causes diseases in humans and animals [1]. In humans, it is frequently associated with meningitis, neonatal sepsis and may also affect immunocompromised adults and the elderly [2]. *S. agalactiae* is responsible for the most fatal bacterial infections in human newborns [3]. In fish, the pathogen causes meningoencephalitis and septicemia worldwide, in both fresh-water and salt-water species [4, 5]. Consumption of fish has been associated with an increased risk of colonization by *S. agalactiae* serotypes Ia and Ib in people [6]. *S. agalactiae* continues to be a major cause of subclinical mastitis in dairy cattle, which is the dominant health disorder affecting milk production in the dairy industry, and is responsible for substantial financial losses in that industry worldwide [7].

*S. agalactiae* is of great medical and veterinary importance due to a high social and economic impact [8], together with the incidence of diesase in different hosts [9]. The incidence of invasive infections unrelated to pregnancy in human adults and animals is increasing worldwide [10]. Therefore, further studies in the area remains necessary. Since the 1990s, serotype V emerged in the United States as the most frequent *S. agalactiae* serotype causing invasive disease in nonpregnant adults [11]. Nowadays, other serotypes including Ia and III have also been recognized in different countries as significant cause of invasive diseases [12]. Comparative genomic studies among several *S. agalactiae* strains of the different serotypes will contribute to a better understanding of the biological complexity of the species. One such reason drove this study for genome sequencing, assembly and annotation of the GBS85147 *S. agalactiae* serotype Ia and Sequence Type 103 (ST-103) strain. The pathogenic potential of this human isolate obtained from the oropharynx of an asymptomatic female patient suffering of various recurrent pharyngitis episodes has been increasingly observed in different investigations [13–16]. From six *S. agalactiae* strains of Ia, III and V serotypes, only serotype Ia, including strain GBS85147, was capable of triggering a respiratory oxidative burst during adherence to the surface of activated macrophages. This activity was demonstrated by NADPH-oxidase activation within phagocytic vacuoles, indicating a high ability of strain GBS85147 isolated from an asymptomatic patient to survive in aerobic stress conditions. Moreover, the invasive potential of strain GBS85147 was also demonstrated by bacterial adherence, invasion and survival (24 h) in the intracytoplamatic environment of endothelial cells. Moreover, the detection of sialic acid in bacteria is limited to a few examples, which, strikingly, are all pathogenic, including *S. agalactiae*. Similar to serotypes III and V, sialic acid residues were also detected on the surface of serotype Ia GBS85147 strain. These findings reinforce the pathogenic potential of *S. agalactiae* GBS85147 strain by its ability to interfere specifically with opsonic components due to inhibition of the alternative complement pathway by serum deficient in a specific antibody [16, 17].

## Organism information

### Classification and features

**Fig. 1:**
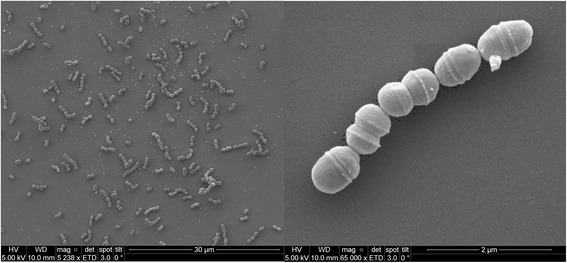
SEM photomicrograph of *S. agalactiae* Gbs85147. Scanning electron microscopy of *Streptococcus agalactiae* strain GBS85147 grown in liquid media after 8 h. Scale bars, 2 and 30 μm, respectively

*S. agalactiae* is a Gram stain-positive, non-sporulating bacterium having a spherical shape with dimensions ranging from 0.2 to 1.0 microns [10] in diameter. On solid medium, *S. agalactiae* may form short chains or may form groups of double cocci. In liquid cells, the microorganism can form long chains (Fig. 1). The bacterium is a facultative anaerobe, catalase and oxidase negative, and is capable of lactic acid fermentation [18]. Lancefield identified the group B antigen, a peptidoglycan-anchored antigen (rhamnose, galactose, N-acetylglucosamine, and glucitol), that defines the *S. agalactiae* species [19, 20].

A capsular polysaccharide antigen is used to classify *S. agalactiae* strains into serotypes [21]. The structure of the CPS is determined by genes encoding the enzymes responsible for its synthesis [22]. Serotype classification is based on the capsular antigen differences detected by PCR or by immunodiffusion techniques [23]. Currently, ten serotypes have been described (Ia, Ib, II, III, IV, V, VI, VII, VIII, IX); serotype IX was identified in 2007 [24]. In some strains, serotype identification is not possible due to the absence of the polysaccharide, caused by a mutation in the capsular genes [25]. The high degree of variation in the capsular structure is related to the virulence of different strains of *S. agalactiae* [26]. Those variations in the capsular structure may also explain its infection of unusual hosts such as camels, dogs, horses, seals, chickens, dolphins, cats, hamsters, frogs, and monkeys [9].

**Fig. 2:**
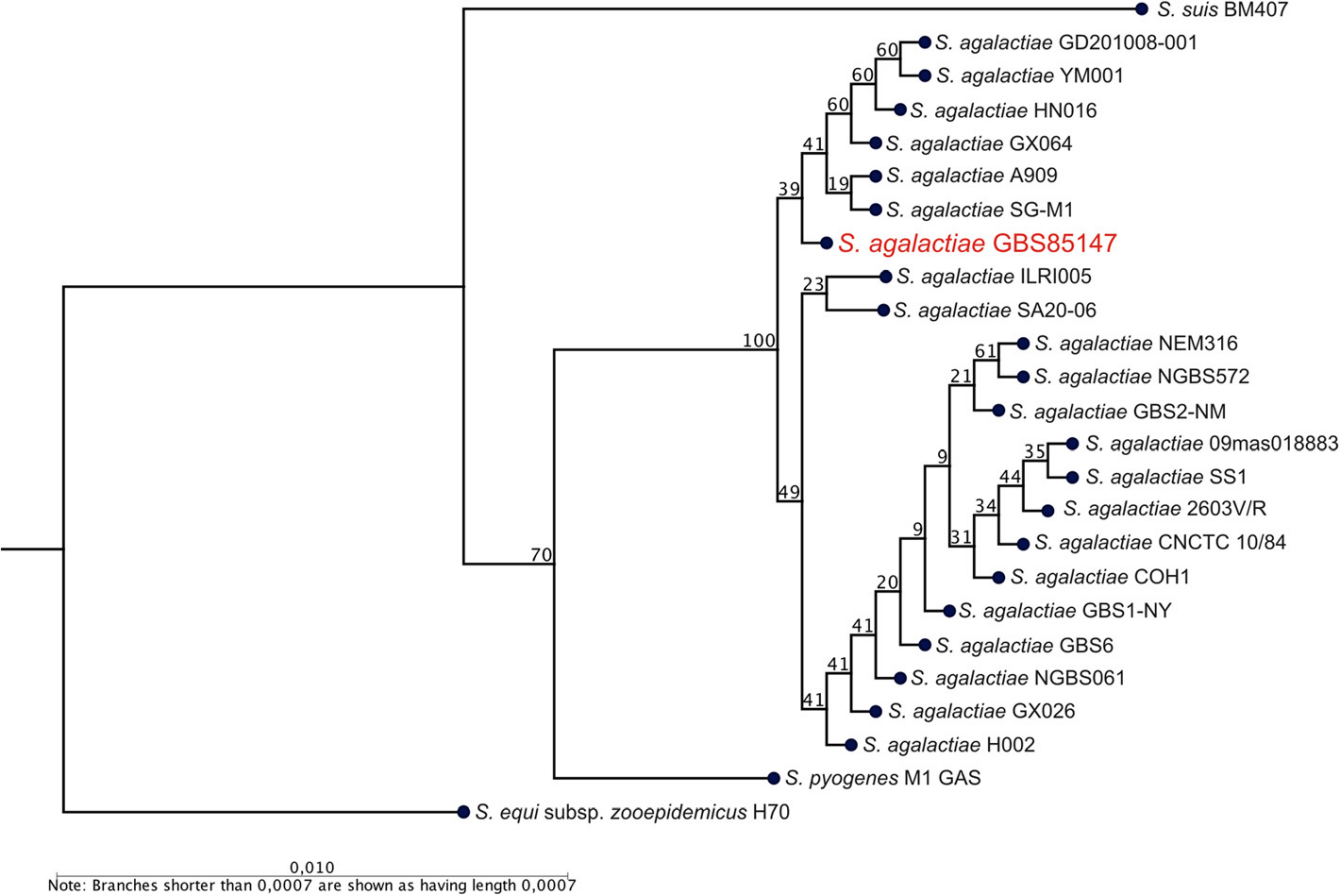
Phylogenetic tree of *S. agalactiae* GBS85147 strain representing its position relative to other type strains. The phylogenetic tree was generated using *S. agalactiae* GBS85147 strain, 21 strains of *Streptococcus agalactiae*, and 3 strains from the genus *Streptococcus* as outgroup strains available at GenBank. The align and tree were constructed with CLC Genomic Workbench using Neighbor Joining method and Jukes-Cantor measure of nucleotide distance with 1000 bootstrap replications

A phylogenetic analysis was performed using, in total 25 different strains, including *S. agalactiae* GBS85147 strain, plus 21 strains of *Streptococcus agalactiae**,* and 3 strains from the genus *Streptococcus**,* as outgroup strains, available at GenBank. The 16S rRNA genes, with mean length of 1,526 ± 50 bp, were aligned with CLC Genomics Workbench (Qiagen, USA). The phylogenetic tree was generated in the same software with the Neighbor Joining method and Jukes-Cantor measure of nucleotide distance with 1,000 bootstrap replications. The phylogenetic tree demonstrates the placement of GBS85147 strain with other closely related strains from the same species, forming a specific clade in 100 % of replications, while it remained distant from the *Streptococcus* spp. *equi, suis,* and *pyogenes* (Fig. 2). All 16S rRNA genes found on assembled contigs were in an equal form. Through this data we observed no contamination and evidence of correct identification of GBS85147 strain. Other features of the strain can be viewed in Table 1.

**Table 1:** Classification and general features of *S. agalactiae* strain GBS85147 – MIGS [27]

MIGS ID	Property	Term	Evidence code^a^
	Classification	Domain *Bacteria*	TAS [47]
		Phylum *Firmicutes*	TAS [48]
		Class *Bacilli*	TAS [49]
		Order *Lactobacillales*	TAS [50]
		Family *Streptococcaceae*	TAS [51]
		Genus *Streptococcus*	TAS [52]
		Species *Streptococcus agalactiae*	TAS [53] [54]
		Strain GBS85147	IDA
		Sorotype Ia	IDA
	Gram stain	Positive	IDA [10]
	Cell shape	Coccus-shaped	IDA [18]
	Motility	Non-motile	IDA [10]
	Sporulation	Non-sporulating	IDA [10]
	Temperature range	Mesophile	TAS [54]
	Optimum temperature	37 °C	IDA
	pH range; Optimum	5.4 – 9.4; 7.4	IDA
	Carbon source	Not Reported	NAS [19]
MIGS-6	Habitat	Human pharynx	IDA [8]
MIGS-6.3	Salinity	4.0 to 6.0 %	IDA [54]
MIGS-22	Oxygen requirement	Facultative anaerobe	IDA [18]
MIGS-15	Biotic relationship	Symbiotic	IDA [8]
MIGS-14	Pathogenicity	Pathogen	IDA [13]
MIGS-4	Geographic location	Rio de Janeiro, Brazil	
MIGS-5	Sample collection time	Not reported	IDA
MIGS-4.1	Latitude	Not reported	IDA
MIGS-4.2	Longitude	Not reported	IDA
MIGS-4.4	Altitude	Not reported	IDA

^a^Evidence codes - *IDA* inferred from direct assay, *TAS* traceable author statement (i.e., a direct report exists in the literature), *NAS* non-traceable author statement (i.e., not directly observed for the living, isolated sample, but based on either a generally accepted property for the species or anecdotal evidence). These evidence codes are from the Gene Ontology project [55]

## Genome sequencing information

### Genome project history

*S. agalactiae* strain GBS85147, taken from a human oropharynx, was isolated in the Laboratory of Molecular Biology and Physiology of Streptococci in the city of Rio de Janeiro, RJ, Brazil. The genome was sequenced, assembled and annotated at the Laboratory of Cellular and Molecular Genetics in collaboration with the National Reference Laboratory for Aquatic Animal Diseases, Ministry of Fisheries and Aquaculture, both located at the Federal University of Minas Gerais, Belo Horizonte, Minas Gerais, Brazil. The genome project was deposited to the public database and the complete genome sequence is available in the GenBank under the accession number Genbank ID CP010319. Further, project information and association with MIGS version 2.0 compliance [27], are summarized in Table 2.

**Table 2:** Project information

MIGS ID	Property	Term
MIGS-31	Finishing quality	Finished
MIGS-28	Libraries used	Fragment
MIGS-29	Sequencing platforms	Ion Torrent™ PGM System
MIGS-31.2	Fold coverage	246x
MIGS-30	Assemblers	Mira v3.9.18
MIGS-32	Gene calling method	FgenesB
	Locus tag	GBS85147
	Genbank ID	CP010319
	Genbank Date of Release	05/01/2015
	GOLD ID	
	BIOPROJECT	PRJNA263907
MIGS 13	Source Material Identifier	SAMN03108598
	Project relevance	Medical, Veterinary, Biotechnological

### Growth conditions and genomic DNA preparation

*S. agalactiae* GBS85147 was obtained from the *Streptococcus* bacterial collection of the Laboratory of Molecular Biology and Physiology of *Streptococci*. The sample was grown on 30 mL of brain-heart-infusion broth (BHI-HiMedia Laboratories Pvt. Ltda, India), with shaking for 48 h at 37 °C. Chromosomal DNA was extracted from 30 ml of bacterial culture. Briefly, the culture was centrifuged at 4 °C, 4000 rpm, for 15 min. Cell pellets were re-suspended in 600 μL Tris/EDTA/NaCl [10 mMTris/HCl (pH7.0), 10 mM EDTA (pH8.0), and 300 mMNaCl], placed 2 times in tubes containing lysed cells with Precellys® at rotations of 6500 rpm for 30 s. The DNA was purified using phenol/chloroform/isoamyl alcohol (25:24:1), precipitated using ethanol/NaCl/glycogen (2.5 vol ethanol, 10 % NaCl and 1 % glycogen) and re-suspended in 30 μL MilliQ®. Finally, the DNA was stained using ethidium bromide and visualized in 1 % agarose gel [28].

### Genome sequencing and assembly

Genome sequencing was performed using a fragment library with the Ion Torrent™ Personal Genome Machine System, with 200 bp sequencing kit. The sequencing produced a total of 578,082,183 bp, distributed among 2,973,022 reads, with an average genome coverage depth of 246-fold and a Phred quality greater than or equal to 20 in 91.25 % of bases. *De novo* assembly was performed using Mira v3.9.18 [29]. The assembly resulted in 104 contigs, accounting for 2,032,890 bp and an N50 of 104.996 bp.

Twenty of the contigs obtained were randomly used as query on BlastN+ [30], over NR database to identify the most similar *S. agalactiae* complete genome deposited in GenBank. After that, the contigs were ordered and oriented using the software CONTIGuator v2 [31] with *S. agalactiae*GD201008-001 [32] as a reference genome, generating a pseudo chromosome with 31 scaffolds. The remaining gaps were closed removing overlaps of neighboring contigs and via consensus sequences obtained by mapping the raw data against the reference genome using CLC Genomics Workbench 7.0 (Qiagen, USA) [33] and BlastN. Furthermore, only the consensus data was used to close gaps in the rRNA regions.

### Genome annotation

Structural gene prediction was performed using the FGENESB [34]. To choose a reference, twenty random parts of our genome were used as query on BlastN over the available four *S. agalactiae* genomes on FGENESB. Therefore, using *S. agalactiae* 09mas018883 [35] as reference, the prediction resulted in 1,616 genes. The genome annotation was performed manually with Artemis [36], UniProt databases [37] and Interproscan 5 [38]. During manual annotation, 299 additional genes were added. For the prediction of rRNA and tRNA the software RNAmmer v1.2 [39] and tRNAscan-SE [40] were used, respectively.

## Genome properties

**Fig. 3:**
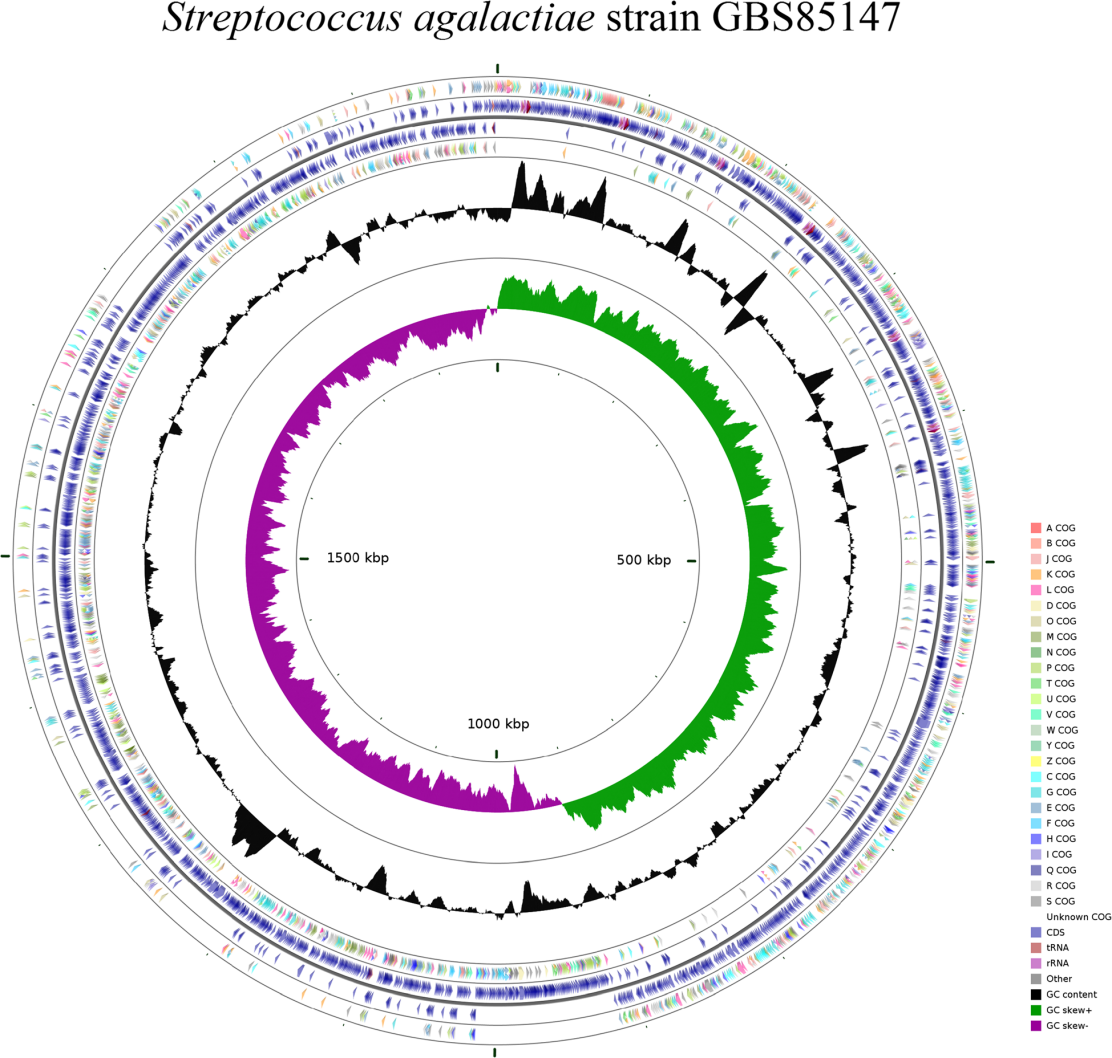
Map of Circular genome generated with CGview comparison tool. In the outermost ring the genes identified by the COG, followed by Blue CDS, tRNAs in orange, rRNAs in pink, other RNAs in gray. In the intermediate ring GC content in black and the innermost ring represents the GC skew + in green and GC skew- in purple

The genome has one circular chromosome with 1,999,151pb, 35.48 % G + C content, a total of 1,998 CDS, including 1,915 protein-coding genes, 18 rRNAs, 63 tRNAs and 2 pseudogenes. A circular map of the genome was generated using the CGView Comparison Tool [41], shown in Fig. 3. Genome statistics are summarized in Tables 3 and 4. Functional analysis using the COG base showed that approximately 27 % of the genes do not have any described function, which consists in the sum of genes with unknown functions (7.69 %) and genes that were not found in the database (19.97 %).

**Table 3:** Genome statistics

Attribute	Value	% of Total
Genome size (bp)	1,996,151	100.00
DNA coding (bp)	1,804,165	90.38
DNA G + C (bp)	708,380	35.48
DNA scaffolds	1	100.00
Total genes	1,998	100.00
Protein coding genes	1,915	95.84
RNA genes	81	4.05
Pseudogenes	2	0.1
Genes in internal clusters	26	1.30
Genes with function prediction	1,713	85.73
Genes assigned to COGs	1,564	78.27
Genes with Pfam domains	1,651	82.63
Genes with signal peptides	111	5.55
Genes with transmembrane helices	511	25.57
CRISPR repeats	1

**Table 4:** Number of genes associated with general COG functional categories

Code	Value	% age	Description
J	144	6.63	Translation, ribosomal structure and biogenesis
A	0	0.00	RNA processing and modification
K	115	5.29	Transcription
L	97	4.46	Replication, recombination and repair
B	1	0.05	Chromatin structure and dynamics
D	20	0.92	Cell cycle control, Cell division, chromosome partitioning
V	36	1.66	Defense mechanisms
T	61	2.81	Signal transduction mechanisms
M	106	4.88	Cell wall/membrane biogenesis
N	7	0.32	Cell motility
U	30	1.38	Intracellular trafficking and secretion
O	58	2.67	Posttranslational modification, protein turnover, chaperones
C	52	2.39	Energy production and conversion
G	173	7.96	Carbohydrate transport and metabolism
E	155	7.13	Amino acid transport and metabolism
F	74	3.41	Nucleotide transport and metabolism
H	50	2.30	Coenzyme transport and metabolism
I	49	2.25	Lipid transport and metabolism
P	102	4.69	Inorganic ion transport and metabolism
Q	23	1.06	Secondary metabolites biosynthesis, transport and catabolism
R	219	10.08	General function prediction only
S	167	7.69	Function unknown
-	434	19.97	Not in COGs

The total is based on the total number of protein coding genes in the genome

## Insight from the genome sequence

**Fig. 4:**
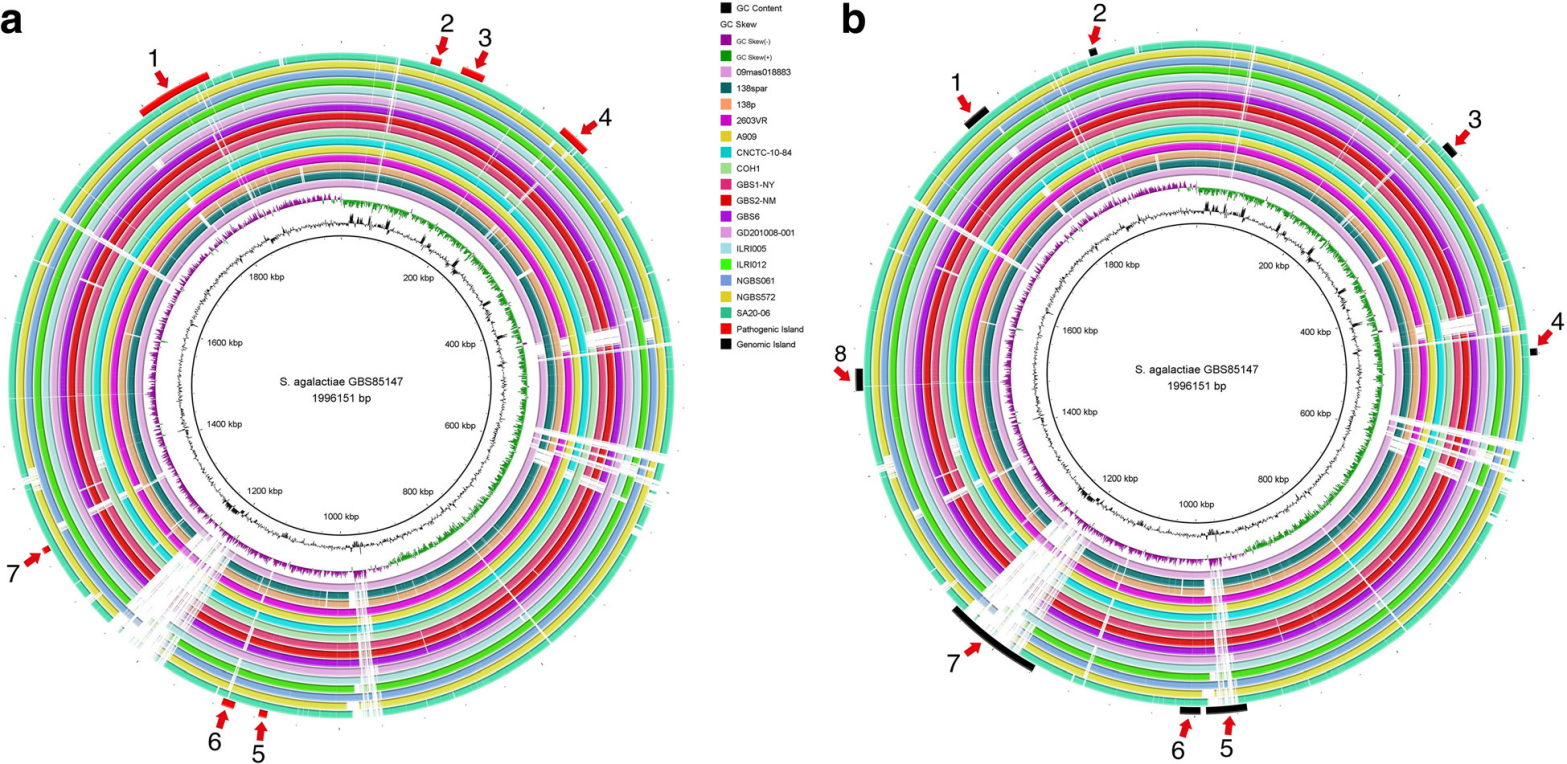
**a**. Predicted pathogenic and genomic islands and comparative visualization. Representation of the seven pathogenic islands predicted by Gipsy software comparing the *S. agalactiae* GBS85147 strain against the 16 complete genomes of the *S. agalactiae* species obtained from the NCBI database. **b**. Representation of the eight genomic islands predicted using the same software by comparing the *S. agalactiae* GBS85147 strain against the 16 complete genomes of the *S. agalactiae* species obtained from the NCBI database. From the inner to outer ring (black) we used the genome of *S. agalactiae* GBS85147 strain as a reference, followed by GC - (purple) and GC + (green) content, the strains of *S. agalactiae* 09mas018883 [35], 138P [56], 138spar [57], 2603 V/R [58], A909 [59], CNCTC10/84 [60], COH1 [61], GBS1-NY [62], GBS2-NM [62], GBS6 [62], GD201008-001 [32], ILRI005 [63], ILRI112 [63], NGBS061 [64], NGBS572 [64] and SA20-06 [65] respectively. The last external ring in 4A display the pathogenic islands while the last external ring in 4B display the genomic islands, respectively

To predict pathogenic islands, GIPSy software [42] was used. GBS85147 strain was compared against 16 complete strains of the same species found at GenBank. BRIG software [43], was used to view the circular structure of pathogenic Islands and the genome strains. Figure 4a represents the seven predicted pathogenicity islands; especially pathogenicity island 4 that consists of six genes, representing four conserved hypothetical proteins whereas two of them are not conserved in all strains. The first one is “Streptokinase”, an enzyme usually secreted by *Streptococcus* species and has a high therapeutic potential to combat thrombolysis, also currently used to combat heart attack and pulmonary embolism [44]. The second “Glycine betaine/proline transport system”, makes part of the glycine betaine transport complex [45]. Glycine is involved in the formation of the peptidoglycan cell wall of Gram-positive bacteria and also helps in securing external cell structures [46], indicating that the bacteria have evolved abilities to survive the stress within the host cells, becoming more resistant to the intracellular environment. Figure 4b (Additional file 1) shows eight genomic islands of unknown classification. This result indicates that Gipsy recognized the region as a probable genomic island, but could not identify it. An in-depth analysis of the genes present in this island revealed that much of the genes products hypothetical proteins, highlighting an importance of conducting further studies for genes present in this region in order to better characterize their functions.

## Conclusion

The genome sequence of *S. agalactiae* GBS85147, obtained using the Ion Torrent PGM platform with approximately 246-fold coverage, was completely finished, manually annotated, its putative pseudogenes manually curated and the resulting genome file deposited in NCBI. After manual annotation of CDSs, the function of 1,713 (85.73 %) genes was identified and, after frameshift manual curation, only two pseudogenes remained. The final size of the genome is ~2 Mb with G + C content of 35.48 %, consistent with the genomes of other strains of the *S. agalactiae* species.

The complete genome of GBS85147, the first isolate of oropharynx of an adult patient in Brazil, can help in further understanding the dissemination of this disease, and improve the identification of genes that allow the *S. agalactiae* serotype Ia to trigger the respiratory oxidative burst during adherence to the surface of activated macrophages. Furthermore, our data may become valuable to future comparative studies with other *S. agalactiae* strains of different serotypes in order to explore their virulence determinants, evolutionary relationships and the genetic basis of host tropism in *S. agalactiae*.

## Additional file

Additional file 1: Strain ID Summary. (DOC 26 kb)

## Abbreviations

CPS: Capsular polysaccharides
GBS: Group B *Streptococcus*
NT: Not type
PGM: Personal genome machine
