# Supplementary material for: Complete genome sequence of Streptococcus agalactiae strain GBS85147 serotype of type Ia isolated from human oropharynx
Source: Stand Genomic Sci. 2016 Jun 3;11:39. doi: 10.1186/s40793-016-0158-6 (PMC4891928; doi:10.1186/s40793-016-0158-6)
Supplement: Additional file 1: — Strain ID Summary. (DOC 26 kb) [file 40793_2016_158_MOESM1_ESM.doc]

**Strain ID Summary**

| Strain ID | Summary |
| --- | --- |
| CNCTC10 | Collection Code: CNCTC  Collection Name: Czech National Collection of Type Cultures  Institution: CNCTC (Czech National Collection of Type Cultures) - null  Strain ID: CNCTC10 |
